# Pandemic treaty textual analysis: ethics and public health implications

**DOI:** 10.1093/pubmed/fdaf040

**Published:** 2025-04-16

**Authors:** Emma M R Anderson, Elizabeth Fenton, John A Crump

**Affiliations:** Bioethics Centre, Dunedin School of Medicine, Division of Health Sciences, University of Otago, Level 1/71 Frederick Street, Dunedin 9016, New Zealand; Bioethics Centre, Dunedin School of Medicine, Division of Health Sciences, University of Otago, Level 1/71 Frederick Street, Dunedin 9016, New Zealand; Centre for International Health, University of Otago, 55 Hanover Street, Dunedin 9016, New Zealand

**Keywords:** COVID-19, ethics, government and law

## Abstract

**Background:**

The World Health Organization’s convention, agreement or other international instrument on pandemic prevention, preparedness, and response, often referred to as the ‘pandemic treaty’, was established with principles to guide implementation. The treaty’s underlying ethic was cosmopolitan in intent, emphasizing equal value of all people with obligations stemming from shared humanity.

**Methods:**

The principles of the working draft of 13 July 2022 and the proposed agreement of 22 April 2024 were compared by textual analysis for content and sequence. Changes were analysed using the ethical framework of cosmopolitanism and associated public health implications identified.

**Results:**

Compared with the working draft, the proposed agreement consolidated principles such as solidarity and reduced specific obligations, weakening ethical demands. Sovereignty was elevated to the cardinal principle, while obligations tied to equity and human rights were less specific, reflecting a shift from cosmopolitan intentions and a reduced emphasis on cooperation for shared public health goals.

**Conclusions:**

Changes made through the pandemic treaty negotiation process suggest ethical amnesia, undermining global equity, justice, and solidarity with consequences for public health and pandemic preparedness. Strengthening obligations in the treaty text is essential to embed a collective motivation for cooperation necessary for effective public health before the next pandemic.

## Introduction

The COVID-19 pandemic exposed weaknesses in global health governance, demonstrating that health crises cannot be managed effectively by individual states acting alone.^1^ The International Health Regulations, the primary framework for managing public health emergencies, lacked obligations for cooperation between states, leading to COVID-19 pandemic response failures as states prioritized their own citizens over global needs.^2–6^ The Independent Panel for Pandemic Preparedness and Response (IPPPR) emphasized this shortcoming, stating that a lack of global leadership led to poor coordination and inequitable resource distribution.^7^ For example, COVID-19 vaccine inequity was labelled ‘the biggest moral failure of our times’.^8^ Beyond vaccine access, COVID-19 revealed broader health disparities between high- and low-income countries, as well as among marginalized communities.^9^

The pandemic’s failures suggest an ethical and practical need for cooperative frameworks. In response, many WHO member states and global leaders called for WHO to create a pandemic agreement to strengthen global health equity commitments and cooperation.^6^^,^^10^ The WHO convention, agreement or other international instrument on pandemic prevention, preparedness, and response (WHO CAII), known as the ‘pandemic treaty’, drafted by an intergovernmental negotiating body (INB) of the WHO seeks to improve global cooperation and coordination for future pandemics.

The principles of the treaty, set out in Article three, provide the pandemic treaty’s overarching ethical and normative scaffolding and indicate the commitments and responsibilities of signatories. We suggest that calls for a pandemic treaty from the IPPPR and others, along with the principles outlined in the treaty’s first draft, were guided by a cosmopolitan approach to global health justice, emphasizing respect for human rights, sovereignty, equity, solidarity, and shared responsibility. Broadly, a cosmopolitan approach encourages global cooperation, solidarity, and a focus on obligations that all persons have ‘to those whom we do not know, but whose lives should be of concern to us’.^11^ The cosmopolitan approach is reflected in the stated intention of the treaty, to ‘foster an all-of-government and all-of-society approach’ by ‘greatly enhancing international cooperation’ and working in solidarity to mitigate the societal and economic impact of future pandemics.^6^

The pandemic treaty has undergone multiple revisions since its inception, with changes to its principles reflecting underlying value judgements and the shifting priorities of negotiating parties. We examined whether the pandemic treaty’s cosmopolitan intentions were consistently represented in its principles and how these principles evolved over successive drafts. For the purpose of our study, the ‘working draft’ refers to the first published draft from 13 July 2022 and incorporating contributions from the initial INB meeting.^12^ The ‘proposed agreement’ refers to the latest version available at the time of writing, dated 22 April 2024, developed through two years of INB negotiations with member states and stakeholders.^13^

To evaluate the ethical and public health implications of the revisions to the pandemic treaty, we examined changes to the text between the working draft and the proposed agreement. We assessed the ethical implications of these changes by referring to the cosmopolitan intentions of the treaty and determining the extent to which the changes approach or move away from elements of cosmopolitanism, and also the public health implications of any ethical shifts.

## Methods

This study adopted a cosmopolitanism framework to analyse justice and equity in global health policy. Unlike other justice frameworks that emphasize obligations within specific social or political boundaries, cosmopolitanism situates justice and equity within a global context, recognizing ethical obligations to all individuals, irrespective of nationality, geography, or affiliation. The cosmopolitan framework is particularly relevant for examining pandemic responses and global crises, where risks and consequences are interconnected, and states, institutions, and individuals can significantly impact distant others. Further, a cosmopolitan framework of cooperation and global responsibility promotes action to bring all nations and groups ‘closer to the finish line’ of pandemic response and improved health conditions globally.^14^ A comparative textual analysis of both the content and the sequencing of principles in the working draft and the proposed agreement was conducted to identify, compare, and interpret differences and similarities within a theoretical framework.^15^ This method allows for an understanding of each document’s underlying values, priorities, and the shifts in emphasis or thematic coherence over the drafting process. Given the normative focus of this study, comparative textual analysis enabled us to examine content changes but also the broader ethical and public health implications of these changes in the context of global health policy.

Principles that reflected procedural values rather than ethical ones, such as adhering to international law, were excluded from analysis.^16^ The principles present in both texts were compared textually against the proposed agreement for similarities and differences and grouped into broader thematic clusters of ethical and public health relevance to facilitate identification of overlaps or divergences between drafts. For example, if multiple principles in the working draft discussed human rights, they were grouped and compared with the one human rights principle in the proposed agreement. This categorization captured thematic alignments across the two drafts, creating a foundation for systematically comparing principles across drafts. Principles present in the working draft but omitted in the proposed agreement were identified for analysis.

Direct comparison of the text and the sequential order of each principle relative to other principles was examined, and similarities and differences in text and sequence were highlighted and tabulated. Once changes were identified, we examined for how these shifts might reframe the policy’s ethical priorities. For example, if a principle related to ‘solidarity’ was demoted from a prominent position in the working draft to a later position in the proposed agreement, we interpreted this as a potential reprioritization of this ethical concept in the final policy framework. Textual changes with ethical implications identified by the authors are shown in italics.

The changes were compared to cosmopolitan intention in the creation of the pandemic treaty. This ethical analysis examined the extent to which the principles aligned with the three criteria of cosmopolitism:^17^

Moral worth of persons: That humans are the ultimate unit of moral concern and is not determined by nation, race, ethnicity, or otherwise.
Universalism: That moral worth attaches to all people equally, regardless of nation, race, ethnicity, or otherwise.
Generality: That the duty, or obligations, to recognize moral worth universally applies to all people equally.

These criteria guided our evaluation of whether the principles in the proposed agreement adequately reflect a cosmopolitan ethos. Each principle was analysed for its degree of alignment with these criteria, noting instances where ethical priorities either converged with or diverged from these cosmopolitan criteria, to determine whether cosmopolitanism was met or not met in the pandemic treaty and the extent to which it changed throughout the negotiation process. Finally, the changes were evaluated for public health implications and whether the COVID-19 pandemic public health concerns that precipitated the treaty would or would not be improved by adherence to the proposed agreement principles.

## Results

Textual analysis identified 15 principles in the working draft and five in the proposed agreement. Principles, such as the principles of adhering to scientific evidence and international law, were procedural and remained largely unchanged throughout the negotiation process. The principles of universal health coverage (UHC), One Health, community engagement, and inclusiveness were present in the working draft, but not in the proposed agreement. The principles remaining after comparison across drafts were categorized into sovereignty, solidarity, equity, human rights, and omitted principles from the proposed agreement, presented below. Table 1 shows the procedural and omitted principles, as well as direct textual comparison of the selected principles of ethical and public health relevance in the working draft of the pandemic treaty and the proposed agreement.

**Table 1:** Direct textual comparison of pandemic treaty principles of the working draft and the proposed agreement.

Principle	Working Draft	Proposed Agreement
Sovereignty	‘(10) Sovereignty—States have, in accordance with the Charter of the United Nations and the principles of international law, the sovereign right to determine and manage their approach to public health, notably pandemic prevention, preparedness and response pursuant to their own policies, and the responsibility to ensure that activities within their jurisdiction or control do not cause damage to other States and their peoples’ (p. 8)	‘1. the sovereign right of States to adopt, legislate, and implement legislation, within their jurisdiction, in accordance with the Charter of the United Nations, the WHO Constitution and the principles of international law, and their sovereign rights over their biological resources’ (p. 6)
Solidarity	(6) Transparency—International action to prevent and prepare for pandemics depends on coordinated, timely and transparent sharing of information, data, and other factors necessary to ensure countries are able to carry out a robust response, for which parties are accountable, through a whole-of-government and whole-of-society approach, based on and guided by the best-available science (7) Accountability—Effective global response to pandemics requires high levels of collective capacity by all countries. All parties are accountable for strengthening and sustaining their health systems’ capacities and public health functions in order to collectively strengthen, support and sustain global prevention, preparedness, and response capacities. (8) Solidarity—Intensified international cooperation, based on a set of specific obligations for parties (especially, but not limited to, obligations from developed to developing countries) is required to prevent, prepare for, respond to, and recover from pandemics (9) Shared but differentiated responsibilities and capabilities—Full consideration and prioritization are required of the specific needs and special circumstances of developing country parties, especially those that (i) are particularly vulnerable to adverse effects of pandemics, (ii) do not have adequate conditions to respond to pandemics, and (iii) would have to bear a disproportionate or abnormal burden (12) Inclusiveness—The engagement with and participation of all relevant stakeholders and partners, consistent with relevant and applicable international and national guidelines, rules, and regulations (including those relating to conflicts of interest) are fundamental to empowering communities and achieving the objective(s) of this WHO CAII	‘5. solidarity with all people and countries in the context of health emergencies, inclusivity, transparency, and accountability to achieve the common interest of a more equitable and better prepared world to prevent, respond to and recover from pandemics, recognizing different levels of capacities, and capabilities’ (p. 7)
Equity	‘(4) Equity—A fair, equitable, effective, and timely response to pandemics requires ensuring fair access to affordable pandemic response products, among and within countries, including between groups of people, irrespective of their social or economic status’ (p. 7)	‘4. equity as a goal and outcome of pandemic prevention, preparedness and response, striving for the absence of unfair, avoidable, or remediable differences among and between individuals, communities, and countries’ (p. 7)
Human rights	‘(1) The right to health—The enjoyment of the highest attainable standard of health, defined as a state of complete physical, mental, and social well-being, is one of the fundamental rights of every human being without distinction of age, race, religion, political belief, economic, or social condition’ ‘(3) Respect of human rights—The implementation of the WHO CAII will be with full respect for the dignity, human rights, and fundamental freedoms of persons’ (13) Gender equality—Pandemic prevention, preparedness, and response will take into account the specific needs of women and girls, using a country-driven, gender-responsive, participatory, and fully-transparent approach (14) Nondiscrimination and respect for diversity—The impact of pandemics should not impede the enjoyment of the highest attainable standard of health without distinction of race, religion, political belief, economic, or social condition (15) Rights of vulnerable populations—Nationally determined and prioritized actions will take into account vulnerable people, places, and ecosystems. Indigenous populations, refugees, migrants, persons with disabilities, children, and adolescents, for example, may be particularly impacted by pandemics, owing to social and economic inequities, as well as to legal and regulatory barriers that may prevent them from accessing health services	‘2. full respect for the dignity, human rights, and fundamental freedoms of all persons, and the enjoyment of the highest attainable standard of health of every human being’
Omitted principles	(2) Universal health coverage—The WHO CAII will be guided by the goal of achieving universal health coverage as an overarching principle to promote health and well-being for all at all ages (5) One Health—Multisectoral actions that recognize the importance of animal health, human health, and environmental health working together to achieve better public health outcomes (11) Community engagement—As communities are a cornerstone of health, effective, and appropriate pandemic prevention, and preparedness require sustained community engagement efforts that make communities more likely to trust governments in times of vulnerability and uncertainty, such as pandemics, and thereby play a central role that is fundamental to pandemic response (12) Inclusiveness—The engagement with and participation of all relevant stakeholders and partners, consistent with relevant and applicable international and national guidelines, rules, and regulations (including those relating to conflicts of interest) are fundamental to empowering communities and achieving the objective(s) of this WHO CAII	
Procedural principles		3. Full respect of international humanitarian law for effective pandemic prevention, preparedness, and response; 6. The best available science and evidence as the basis for public health decisions for pandemic prevention, preparedness, and response

### Sovereignty

The prominence of sovereignty was promoted in successive drafts from tenth to first. Limits to sovereign power were removed in consecutive drafts, impacting the cosmopolitan element of generality. For example, the working draft included a statement of the obligation of states ‘to ensure that activities within their jurisdiction or control do not cause damage to other States and their peoples’, a statement not present in the proposed agreement.^12^ Furthermore, sovereign rights were strengthened, illustrated by the addition to the proposed agreement of states’ ‘sovereign rights over their biological resources’^13^ (Table 2). The public health implications of these changes are towards increased state self-interest and away from global public health coordination.

**Table 2:** Comparing the working draft and proposed agreement principles to the three elements of cosmopolitanism.

	Moral worth of humans	Universalism	Generality
Working draft	Equity: *‘*among and within countries, including *between groups of people*, irrespective of their social or economic status’ The right to health: ‘fundamental rights of *every human being*’ Respect of human rights: ‘full respect for the dignity, human rights, and fundamental freedoms of *persons*’ Non-discrimination and respect for diversity: ‘should not impede the enjoyment of the highest attainable standard of health *without distinction’* Universal health care: ‘promote health and well-being *for all* at all ages’ One Health: human health: ‘recognize the importance of animal health*, human health* and environmental health’ Community engagement: ‘*communities* are a cornerstone of health’	Solidarity: ‘with *all people* and countries’ The right to health: ‘fundamental rights of *every human being’* *Universal Health Care:* promote health and well-being *for all at all ages*	Sovereignty: ‘the *responsibility* to ensure that activities within their jurisdiction or control *do not cause damage to other States and their peoples*’ Transparency: ‘through a *whole-of-government and whole-of-society approach’* Accountability ‘requires high levels of *collective capacity by all countries’* Solidarity: ‘Intensified international *cooperation’* Shared but differentiated responsibilities and capabilities: ‘Full *consideration and prioritization* are *required* of the specific needs and special circumstances’ Universal Health Coverage: ‘The WHO CAII *will be guided by the goal* of achieving universal health coverage’ One Health: ‘*Multisectoral actions* that recognize the importance of animal health, human health, and environmental health *working together to achieve better public health outcomes*’ Community engagement*: ‘require* sustained community engagement efforts’ Inclusiveness: ‘*The engagement with and participation of all relevant stakeholders and partners…are fundamental* to empowering communities *and achieving the objective(s)* of this WHO CAII’
Proposed agreement	Equity ‘among and between *individuals, communities and countries*’ Human rights: ‘highest attainable standard of health of *every human being*’	Solidarity ‘with *all people* and countries’ Human rights ‘full respect for the dignity, human rights, and fundamental freedoms of *all persons*’	Limits of sovereignty removed Obligations removed

### Solidarity

In the proposed agreement, the principle of solidarity combined five principles from the working draft: transparency, accountability, shared but differentiated responsibilities, and capacities, inclusiveness, and solidarity. In both the working draft and the proposed agreement, solidarity with ‘all people’ suggested both that persons are the unit of moral concern, and that it was universal, to ‘all’ equally (Table 2).

The principle of generality was met in the working draft but not clearly met in the proposed agreement. The working draft outlined some obligations attached to each principle. For example, in the working draft, transparency is ‘through a whole-of-government and whole-of-society approach’, accountability ‘requires high levels of collective capacity by all countries’ and solidarity is ‘Intensified international cooperation based on a set of specific obligations for parties’ (Table 2).

In the proposed agreement, solidarity came with no associated obligations and was not emphasized among the other principles in the proposed agreement. In the working draft, solidarity was ‘required’. In the proposed agreement, however, the strongest language was to ‘recognize’ a difference in capacities and capabilities. As such, the consolidation of many principles into one diluted each principle and their associated obligations, and the principle became less demanding of states’ obligations towards collective global public health collaboration.

### Equity

Equity remained the fourth principle in both texts. In both the working draft and the proposed agreement, equity was described as holding ‘between groups of people’ and ‘between individuals’, respectively, suggesting that individuals rather than nations are the focus of moral concern (Table 2).

As for generality, the working draft framed equity as a more demanding principle than in the proposed agreement. There was a *requirement* to ensure fair and affordable access to pandemic response products such as vaccines, irrespective of location, or status. The proposed agreement, while maintaining a definition of equity, substantially weakened the associated obligation from ‘*requiring*’ to ‘*striving* for the absence of unfair, avoidable or remediable differences among and between individuals, communities, and countries’, thus impeding on collective global public health efforts to reduce inequity.

### Human rights

In the working draft, human rights was the first principle, and human rights were reflected across five other principles. The proposed agreement placed human rights as the second principle, encapsulating the five human rights principles from the working draft.

The moral worth of persons was evident in the working draft in references to the respect of ‘persons’ and non-discrimination ‘without distinction’. Human rights of ‘every human being’ also suggests generality and universalism and was present in both drafts (Table 2). The working draft limited the application of the rights of vulnerable populations to the ‘nationally determined and prioritized actions’, whereas the application in the proposed agreement was broad and applied to all in ‘full respect’ of the rights of persons but had no concrete obligations attached. Consequently, the element of generality cannot be assumed, and the obligation to global public health not solidified.

### Omitted principles

The omission of UHC, One Health, community engagement, and inclusiveness removed some cosmopolitan elements from the proposed agreement. The cosmopolitan element of viewing persons as the unit of moral concern was present in the principles of UHC, One Health, and community engagement which respectively stated the need to ‘promote health and well-being for all at all ages’, the importance of ‘human health’, and asserted that ‘communities are a cornerstone of health’. UHC noted the universal application ‘for all’. The cosmopolitan element of generality was present in each of the omitted principles. The principle of UHC stated that ‘The WHO CAII will be guided by the goal of achieving UHC’, meaning that actions were to be implemented with UHC in mind. The principle of One Health suggested a ‘multisectoral’ duty of ‘working together to achieve better public health outcomes’. The principle of community engagement ‘require[d] sustained community engagement efforts’. The principle of inclusiveness stated that ‘The engagement with and participation of all relevant stakeholders and partners’ was ‘fundamental to empowering communities and achieving the objective(s) of this WHO CAII’.

The omission of key principles in the proposed agreement weakens the intersectoral coordination necessary for effective pandemic prevention and response. For example, the exclusion of UHC limits equitable access to healthcare, and coordination with increasing fragmentation and delaying outbreak detection and control. The absence of One Health undermines collaboration across human, animal, and environmental health sectors, reducing the capacity to address zoonotic disease threats. Community engagement is important for fostering trust and ensuring context-specific interventions, and its omission risks diminishing public cooperation. Similarly, the lack of inclusiveness overlooks the needs of marginalized populations, exacerbating inequities and the engagement of populations most impacted by policy decisions. Collectively, these omissions hinder the complex, multisectoral approach required for effective pandemic preparedness and response. From a public health perspective, the omissions may lead to less coordination across multiple sectors, including the interactions between health systems, communities, animals, and environments. All of which are important for prevention and response to pandemics.

## Discussion

### Main findings of this study

Concerning the three elements of cosmopolitanism, the proposed agreement for a pandemic treaty acknowledges some elements while compromising others. Moral concern of individuals and universalism remained largely intact throughout the negotiating process. However, the element of generality, which captures the obligations of persons to others regardless of their citizenship, has been substantially eroded between pandemic treaty versions. While rationalization and integration of overlapping principles may have been appropriate, the principles listed in the proposed agreement no longer required cooperation between states for the benefit of all. While the principles of equity, human rights, and solidarity were broadly applied, the principle of sovereignty may have entangled them with nationalism. Indeed, it is the elevation of sovereignty to the status of the first principle that is in tension with an obligation to cooperate, suggesting that state interests, which might include economic or security interests, are prioritized over the welfare of all individuals globally. Indeed, the omitted principles suggest a reframing of ethical priority towards the state and away from communities and stakeholders.^18^

The revisions to the pandemic treaty in the proposed agreement represent a regression to the pre-pandemic status quo in which nationalism overrides global public health cooperation and solidarity. For example, objections from the USA cite sovereignty concerns.^19^ This position was also evident in accounts of vaccine nationalism and illustrated the need for a cosmopolitan approach to public health.^20^^,^^21^ While efforts to develop SARS-CoV-2 vaccines succeeded only because of substantial institutional and governmental cooperation, its equitable allocation proved to be an ethical and public health challenge that pitted states against one another and favoured the ability of high-income states to secure advance-purchase agreements and other mechanisms to vaccinate their populations as early as possible.^22^ For example, critical analyses of even an epidemiologically successful COVID-19 response in New Zealand showed that nationalism was leveraged by framing the response as an international competition at odds with the idea of global solidarity.^23^ When the state is the ultimate unit of moral concern, nationals are prioritized, often at the expense of public health globally.^24^ As it stands, these problems that were the impetus for the creation of the pandemic treaty and its ability to address them have been diluted through the negotiation process.

### What is already known in the topic

Many have recognized the ethical challenges in the calls for equitable allocation of the COVID-19 vaccine and the importance of ethics during the COVID-19 pandemic^25^ and in the pandemic treaty.^16^ The proposed agreement’s undemanding obligations have led some to label it the pandemic treaty ‘lite’.^26^^,^^27^ Previous work has emphasized the need for clear, enforceable ethical principles in health treaties. Schaefer *et al*. argued for precise treaty principles directly linked to policy, suggesting that sovereignty and human rights be treated as underlying values rather than explicit ethical principles, flagging the risk that sovereignty would be conflated with nationalism.^16^ Other commentators have noted how the COVID-19 pandemic highlighted that ethical challenges are as important as technical challenges and that the pandemic treaty, and global pandemic preparedness generally, should incorporate actionable ethical statements on equity and solidarity.^28^^,^^29^

### What this study adds

Our study supports previous research indicating that while including sovereignty as a guiding principle and reducing obligations related to equity, solidarity, and human rights may make the pandemic agreement more acceptable to states, the prominence of sovereignty in the proposed agreement suggests it ultimately accommodates, rather than counters, state-centric tendencies it aimed to overcome. Instead of challenging these self-interested behaviours in a ‘cosmopolitan moment’,^30^ the proposed agreement yields to them. Nationalism prioritizes those within a nation at the expense of individuals more broadly. During a global pandemic, prioritizing the nation is ethically problematic and comes with sacrifices for global equity and solidarity, as well as delaying the end of the pandemic.^2^^,^^31^^,^^32^

Overall, while there has been much discussion on the need for ethics, equity, and solidarity in global public health response post-COVID-19,^33^ we argue that the diminished force of the obligations placed on states in the text of successive pandemic treaty drafts is symptomatic of ethical amnesia, or the failure to uphold previously endorsed ethical commitments due to political convenience, that occurs during and after a crisis.^34^^,^^35^ Calls heard during the COVID-19 pandemic to recognize obligations to protect the health of all now appear fleeting and disingenuous, as efforts to protect people from the same inequities of treatment in future global health crises are ignored. While there are predictable challenges in creating an effective pandemic treaty that states can agree on, the revisions to the proposed agreement highlight the ongoing impact of state self-interest on well-intentioned global health goals.^16^^,^^36^^,^^37^ At the opening of the 12th INB, the WHO Director-General, Dr. Tedros Adhanom Ghebreyesus, reminded negotiators ‘to come together to see beyond purely national interest to the global interest, because the world can only be safer if every individual nation is safer’,^38^ highlighting the importance of multilateralism in overcoming global health challenges. Importantly, the pandemic treaty is an opportunity to rebuild trust between higher and lower income countries and to set the behavioural norms and expectations for future global pandemics.^39^

### Limitations of this study

Our exclusion of procedural changes may overlook indirect ethical and public health implications. A triangulated analysis comparing all principles with pandemic treaty articles on governance, implementation, and accountability could reveal implicit ethical or public health impacts. Stakeholder interviews with experts, policymakers, and civil society representatives would further clarify perceptions and interpretations of the changes. Combining these approaches would provide a more comprehensive understanding of the ethical and public health implications that arise from changes to the pandemic treaty.

## Conclusions

Examining revisions to the pandemic treaty through the negotiation process revealed a substantial departure from cosmopolitan cooperation towards protecting state interests. The proposed agreement reduces the number of principles linked to the commitments of cosmopolitanism and weakens the associated obligations imposed on signatories. The proposed pandemic treaty must address the specific inadequacies of public health policy revealed by COVID-19 and establish mechanisms to mitigate similar future challenges. However, the current draft falls short of this objective. We recommend greater scrutiny of the proposed pandemic treaty agreement against its original intent, especially regarding its impact on global public health. If sovereignty is to remain the first principle of the agreement, we recommend strengthening language relating to the obligations of states towards individuals in other states and the obligation of all states to engage in multilateral problem-solving. Strengthening these commitments is crucial to support coordinated and timely responses to public health emergencies, underscoring the ethical obligation of states to mitigate harm not only domestically but also globally. To enhance public health protections, each principle should link to enforceable obligations, such as swift, coordinated actions during health emergencies and equitable sharing of resources, to reduce health risks across borders. While the finalization of a pandemic treaty is essential, it is also important to recognize the public health implications of weakened obligations resulting from these revisions. Such changes may compromise the treaty’s future effectiveness as a tool for coordinated pandemic response. Current outbreaks including highly pathogenic avian influenza, mpox, and Marburg virus disease serve as reminders of the ongoing need for a shift towards cooperative approaches to public health. As negotiations proceed, governments should reevaluate their ethical commitments within the treaty, aiming to strengthen provisions for global health equity, justice, and solidarity to support a more effective and unified response to future pandemics.

## Notes

**Emma M. R. Anderson,** PhD Candidate

**Elizabeth Fenton,** Senior Lecturer

**John A. Crump,** Professor
